# Angina Pectoris

**Published:** 1902-01

**Authors:** Marvin E. Nuckols

**Affiliations:** Richmond, Va., Lecturer on Operative Surgery, University College of Medicine


### ANGINA PECTORIS.*

By MARVIN E. NUCKOLS, M.D., Richmond, Va.,

Lecturer on Operative Surgery, University College of Medicine.

In reviewing all accessible literature on the subject, I find that there is a diversity of opinion as to the exact nature of the condition, some contending that it is but a symptom of some more or less grave vascular lesion, while others claim that it is a disease in itself, always having a definite group of symptoms. No less an authority than William Osler states that it is nothing more than a

* Read before the Richmond Academy of Medicine and Surgery, Nov. 26, 1901.

symptom. Clifford Allbut, a distinguished English physician, holds the exact opposite. When we find such authorities as the above holding opinions entirely different, we should not wonder that the lesser lights have vague and indefinite ideas regarding angina pectoris.

In discussing this condition, I shall of necessity have to deal with a great deal that is now well known to you all; however, in doing so, I shall endeavor to present it in such a way as to arouse some interest, and thus provoke a full discussion of the subject. I wish to be understood in the beginning as not believing in pseudo-angina pectoris; but believe that angina pectoris is angina pectoris, and that the only difference in the cases seen is one of degree; therefore, I eliminate such conditions as the agitations of neurotic women generally due to hysteria, indigestion or intercostal neuralgia, and also certain spasms of tobacco poisoning which differs widely and fundamentally from angina pectoris.

Having then eliminated this, we will take up angina pectoris proper, and we shall find that it is characterized by a very definite group of symptoms. Pain is usually the most prominent, but it is the character of the pain which is of so much value. It cannot be likened to any other pain; being sharp and stabbing, located behind the sternum (not in the heart), radiating upward toward the left shoulder, neck and head, and down the left arm into the fingers, and sometimes into the testicles, which may swell. As I have mentioned, there is no pain in the heart, but there is a feeling of depression or of something lacking in this region. The peculiarity of this pain is that it is accompanied by a feeling of constriction about the chest and neck likened to the pressure of a vise. Balfour decribes it as a feeling as if a mailed hand grasped the chest and squirted through its fingers flashes of agony. This is often accompanied by convulsive movements of the muscles of the whole body, but particularly of the chest, neck and arm.

The expression is extremely anxious, manifesting an indescribable fear of impending dissolution. The attitude is one of terror-stricken stillness. The body is covered with beads of sweat, and the extremities are cold and clammy. Strange to say, the pulse is, as a rule, unchanged. We have no dyspnea like that so often seen in heart disease, though respiration may be increased in frequency, and during severe paroxysms temporarily suppressed. Pain is sometimes entirely absent, or may begin in the hand and extend upward. In these cases the restrosternal pain is not so severe.

The indescribable fear of impending death is never absent. The preceding symptoms are the usual symptoms of an ordinary attack which may last only a few seconds or a few minutes, to be followed by others with increasing frequency.

The attacks are brought on by some unusual or violent exercise, though they may come on while in bed during the night without any apparent cause. As to the actual cause of angina, very little is definitely known. It is certainly not always due to heart disease, as is abundantly proved at the bedside and at post-mortems. A fatal case is reported by T. E. Bullard, which he saw in one hundred and five paroxysms of true angina, during the last of which the patient died. There were no evidences during life or after death of any organic change. Whatever constitutes angina pectoris, this fact is very evident—that a heart organically diseased will fail under repeated attacks of angina, whereas one with no evidence of disease may partially or completely recover.

It is argued by some that a vaso-motor spasm is the immediate cause of angina, bringing on an increased arterial tension and throwing extra work upon the heart. In support of this theory they lay great stress upon the good results derived from the administration of nitrites, but we must remember that the nitrites relax the heart as well as the arteries, and that they possess certain anodyne and antispasmodic properties. The majority of cases of angina show but little if any increase in tension. If angina is due to increase in tension, why don't we always have it in all forms of heart disease accompanied by increase in tension? Is it not possible for pain to cause the increase in tension if there be any? We have abundant evidence that pain can cause increase in tension; as examples of this I might mention kidney colic, cramp in the abdomen, etc.

A theory held by many is that it is a cramp of the heart muscle. Osler states that we may have a localized cramp, or a cramp of some of the muscular fibres of the heart. This theory to me seems altogether untenable, for I cannot conceive of a muscle or some fibres of a muscle undergrowing cramp and still being able to act. It seems to me the power of the muscle is temporarily lost. The quality of the heart muscle to undergo rhythmic contractions is too deeply implanted in its nature to give place to any change, save to either stop completely and permanently, or to prolong diastole. No drug or electrical stimulus can do more than increase force of systole or prolong diastole.

A third theory is that it is nervous in origin. This, to my mind, seems to explain some cases, particularly those with pain beginning in the hand and extending upward, resembling the aura of epilepsy.

A theory accepted by many is that it is a neuritis of the cardiac plexus. In this case pains should always be present, for it is hard to conceive of a neuritis without continouus pain.

A theory that will explain a great many cases is that the cause lies either in an acute or chronic aortitis near the root of the aorta, and as the cardiac plexus of nerves lies in close relation, a neuralgia may be set up secondarily in this plexus. However, let the cause be what it may, we know that the condition is always characterized, with very little variation, by a definite group of symptoms, and that we do not always find manifest organic lesion either of the heart or blood vessels.

It is supposed that the pain is a referred pain, and that it is due in many cases to increased pressure on an inflamed aorta, manifested through the cardiac plexus. One proof of this is seen in mitral regurgitation; if angina has been present, relief is afforded when the compensation fails.

As to how angina kills, there is a difference of opinion. Some say that it is a reflex from inhibition of the vagus. This inhibition is brought about by the violent pain, the weak and degenerated heart and blood vessels being unable to survive the shock.

The treatment may be divided into two parts: (1) During the attack. (2) During the interval. *During the attack,* inhalations of amyl nitrite. If these fail, use chloroform or ether by inhalation, and, as a last resort, give morphia and atropia hypodermically. Use hot applications over the region of the heart.

*During the intervals.* (a) Hygienic and dietetic. Complete change in habits must be advised. Attention should be given to the bowels to see that they act regularly. Avoid excesses of all kinds, particularly of eating and drinking. The diet should be regulated so that no digestive disturbances shall arise. Instructions should be given to avoid excitement and violent exercise.

(b) Medicinal. Nitroglycerine should be given in full and increasing doses. If degeneration of the arteries or any tendency toward rheumatism be evident, iodides should be given in full doses, extending over long periods, but always with an eye to the condition of the stomach.

306 East Main street.

